# Motorcycle Accidents and Their Outcomes amongst Victims Admitted to Health Facilities in Guinea: A Cross-Sectional Study

**DOI:** 10.1155/2020/1506148

**Published:** 2020-06-22

**Authors:** Alexandre Delamou, Karifa Kourouma, Bienvenu Salim Camara, Delphin Kolie, Fassou Mathias Grovogui, Alison M. El Ayadi, Serge Ade, Anthony D. Harries

**Affiliations:** ^1^Centre d'Excellence Africain pour la Prévention et le Contrôle des Maladies Transmissibles (CEA-PCMT), Conakry, Guinea; ^2^Department of Public Health, Gamal Abdel Nasser University, Conakry, Guinea; ^3^Centre National de Formation et de Recherche en Santé Rurale (CNFRSR) de Maferinyah, Forécariah, Guinea; ^4^University of California San Francisco, Bixby Center for Global Reproductive Health, San Francisco, CA, USA; ^5^Faculté de Médecine, Université de Parakou, Parakou, Benin; ^6^International Union Against Tuberculosis and Lung Disease, Paris, France; ^7^Department of Clinical Research Faculty of Infectious and Tropical Diseases, London School of Hygiene and Tropical Medicine, London, UK

## Abstract

**Background:**

Motorcycle road traffic accidents (RTA) constitute an increasing public health challenge with victims more likely to sustain fatal injuries compared with other types of RTA. The aim of this study was to analyze motorcycle RTA-related morbidity and mortality among victims admitted to hospitals in Guinea from 2015 to 2017.

**Materials and Methods:**

This was a cross-sectional study based on hospital records from six districts (Boké, Kindia, Mamou, Faranah, N'Zérékoré, and Siguiri) from January 1, 2015, to December 31, 2017. Bivariate analysis and multivariate logistic regression were used to explore associations between RTA types and mortality.

**Results:**

There were 14,962 RTA victims with motorcycle RTA accounting for 58.3% and other RTA 45.3% of hospital admissions. Overall, motorcycle RTA accounted for 77.7%, with young adults (96.2%) and males (73.5%) more affected when compared to victims of other types of RTA. Median age of motorcycle RTA victims was 23 years (IQR: 17–33 years). Students (29.7%), employees (23.6%), and farmers/housewives (23.3%) were the commonest groups affected by motorcycle RTA. The highest burden of motorcycle RTA occurred in the mining zones (Boké and Siguiri). Wounds (39.2% and 27.3%) and multiple injuries (43.8% and 43.8%) were the commonest types of injury sustained by victims of both motorcycle and other types of RTA, respectively. Motorcycle RTA accounted for 54% of overall deaths. Using multivariate logistic regression analysis, sustaining a motorcycle RTA in N'Zérékoré (AOR: 4.2; 95% CI: 1.6–11.2) and being admitted with mild (AOR: 7.4; 95% CI 2.1–25.8) and heavy or deep coma (AOR: 776.1; 95% CI: 340.2–1770.7) were significantly associated with mortality.

**Conclusions:**

Motorcycle RTA are an important cause of morbidity and mortality in Guinea. Males, young adult users, students, employees, and people from mining zones are the most affected. Better law enforcement and awareness raising among Guinean young adults are promising prevention strategies.

## 1. Introduction

Road traffic accidents (RTA) are a major health problem worldwide, responsible for significant morbidity and mortality [1, 2]. According to the recent Global Status Report on Road Safety (2018), RTA are the current leading cause of death for children and young adults (5–29 years) and the eighth for all age groups [1]. Moreover, about 1.35 million people globally die each year from RTA [1], with an additional 20–50 million people seriously injured or disabled [3]. The World Health Organization estimates that RTA will become the third leading cause of disability in the world by 2030 [4]. Therefore, the Sustainable Development Goal (SDG) target 3.6 has called for initiatives to halve the number of global deaths and injuries from RTA by 2020 [5].

However, achieving this target remains particularly challenging for low- and middle-income countries where 90% of RTA-related deaths occur globally and where there has been a huge increase in the importation and purchase of motorcycles and second-hand cars over the last decade [6, 7]. Motorcycle use is associated with greater risk of serious injury or death, especially in urban settings [8–10]. Use of motorcycles for transportation is more common among the young and economically active population, resulting in a greater accident-related burden among these groups and important economic consequences at the population level [10–12].

In Guinea, the RTA rate has been rising consistently each year and motorcycle involvement in RTA has tripled over a three year period [13]. Contributing factors for increasing motorcycle-related RTA include a lack of public transportation and poor quality of roads and vehicles. In addition, people are increasingly using motorcycles because of their affordability, quick mobility, and ability to access hard to reach areas [7]. Furthermore, there is a growing phenomenon of using motorcycles for income-generating activities in the country. Many motorcycle riders disobey speed limits, lack the appropriate driving competencies, overload the motorcycle with more passengers than recommended, and rarely wear helmets [14]. With increasing economic accessibility of motorcycles in Guinea, the number of motorcycle-related RTA and associated injuries and deaths is expected to increase if appropriate preventive actions are not implemented.

Documenting the current burden of each type of RTA and its attributed morbidity and mortality is important for guiding priority actions to decrease RTA-related consequences and understanding their impact. Thus, the present study was undertaken to generate evidence on the burden of motorcycle-related RTA and its consequences in Guinea by analyzing their morbidity and mortality among victims admitted to hospitals in Guinea from 2015 to 2017.

## 2. Materials and Methods

### 2.1. Study Design and Period

This was a cross-sectional study based on health facility records. The data used covered the period from January 1, 2015, to December 31, 2017.

### 2.2. Setting

Guinea is located in West Africa with an estimated population of 12 million inhabitants in 2018 with the majority living in rural areas (64%) [15].

The country comprises 33 districts within eight administrative regions. The public health system is divided into three levels: the primary level which includes 413 rural and urban health centers; the secondary level comprises 7 regional hospitals, 26 district hospitals, and 8 communal health centers; and the tertiary level comprises three national hospitals [16]. The private sector includes 11 polyclinics and 33 medical and surgical clinics. The total length of the road network in the country is about 44,000 km with only 2,220 km (5%) paved with asphalt [16].

The 2016 statistical yearbook of the country reported that seven urban districts (Boké, Kindia, Kankan, N'Zérékoré, Mamou, Faranah, and Siguiri) and the capital city (Conakry) bear the highest burden of RTA in the country [16]. Therefore, eight health facilities from six urban districts (Boké, Kindia, N'Zérékoré, Mamou, Faranah, and Siguiri) constituted our study sites because they have an emergency ward and their data had appropriate information on the primary variable of interest (type of RTA). The selected health facilities included five regional hospitals, one district hospital, and two private clinics, and the population of these districts accounted for 40% (4, 776, 171 habitants) of the national population [15].

### 2.3. Study Population

We included all RTA victims admitted to the selected public and private health facilities over the period January from 1, 2015, to December 31, 2017.

### 2.4. Data Sources and Measures

Data were collected from the emergency unit and hospital ward registers of the eight health facilities. Information from the different registers was entered into a standardized Excel spreadsheet (version 2016). Data were collected by a team of 11 final year medical students, supervised by the study principal investigator (KK), from April to August 2018.

Sociodemographic characteristics captured included age group (child = <15 years, youth = 15–24 years, adults = 25–64 years, and elderly = 65–99 years), sex, occupation, and type of road user (pedestrian, car passenger, car driver, or motorcyclist (drivers) and motorcycle passengers). Clinical characteristics captured included type of injury (not injured, traumatic brain injury, wound, fracture, and two or more injuries), Glasgow Coma Score (classified as normal = 15, mild coma = 14–10, heavy coma = 9–7, or deep coma = 6–3), and death (yes or no). Type of RTA was designated as involving a motorcycle or others (vehicle or bicycle).

### 2.5. Analysis

Data were cleaned in Excel before exporting into the STATA 15 software (Stata Corporation, College Station, TX, USA) for analysis. We summarized the data using descriptive statistics. We excluded 41% of RTA victims because they were missing data on the primary variable of interest (type of RTA). We compared sex, age, and occupation of those excluded from our analytic sample with those included using the chi-square test for categorical variables and the Student's *t*-test for continuous variables. We found that the groups were similar in terms of sex, age, and nearly all of the eight occupations; the only characteristic that differed significantly between the groups was the proportion of drivers (*P*=0.0398). Within our analytic sample, sociodemographic and clinical characteristics of motorcycle-related RTA versus other RTA were compared using the chi-square test for categorical variables and the Student's *t*-test for continuous variables. Logistic regression was carried out to adjust for potential confounding factors on the outcome of interest (death). Multicolliniarity was checked first, and then a final logistic model was fitted using the stepwise approach. The candidate variables for inclusion in the model were those identified in the literature [13, 17–20]. The results were reported using odds ratios (OR) with 95% confidence intervals (CI), both crude OR(COR) and adjusted OR(AOR).

## 3. Results

Data on a total of 14,962 RTA victims were captured over the study period, with motorcycles accounting for 58.3% (11, 620/25, 662) and other RTA 45.3% (3, 339/25, 662) of hospital admissions in the six districts.

### 3.1. Sociodemographic Characteristics of RTA Victims

The sociodemographic characteristics of RTA victims admitted to target health facilities during the study period are shown in Table 1 by the RTA type. Compared to victims of other RTA, victims of motorcycle RTA were more likely to be male (73.5% vs. 63.6%) and youth (53.4% vs. 41.3% children or youth; median age 23 years (IQR 17–33). Occupational distribution differed significantly by the RTA type: the commonest occupational groups involved in motorcycle RTA were students (29.7%), employees (23.6%), and farmers/housewives (23.3%), while employees (24.9%) and farmers/housewives (22.2%) represented the most affected group for other types of RTA. Regarding the type of road users, motorcyclists were predominant (50.7%) among motorcycle RTA victims, whereas car passengers represented the majority (89.3%) of victims of other types of RTA. In each of the six districts, RTA involving motorcycles were more frequent than all other types of RTA. Across the three years of the study, there was a declining trend in the number of motorcycle RTA from 4,527 cases (78.7% of all motorcycle RTA cases) in 2015 to 3,929 cases (78.0%) and 3,167 (75.8%) in 2016 and 2017.

### 3.2. Clinical Characteristics of RTA Victims

Two or more injuries and wounds were the commonest type of injury among victims of both motorcycle and other types of RTA (43.8% and 43.8%, *P* < 0.001) and (39.2% and 27.3%, *P* < 0.001), respectively (Table 2). Though the proportion of victims with wounds was significantly higher among motorcycle RTA as compared to other types of RTA, the proportion of victims with two or more injuries was similar in both types of RTA. However, the number of victims of motorcycle RTA who sustained two or more injuries was about four times higher than that of other RTA (5,091 vs. 1,461). Victims in mild coma during hospital admission represented 3.0%, and those in heavy or deep coma represented 1.4% among victims of motorcycle RTA compared to 2.3% and 3.6%, respectively, among victims of other types of RTA (*P* < 0.001) (Table 2).

Overall, 4.4% of RTA victims died; mortality was 1.1% amongst motorcycle RTA victims compared with 3.3% amongst other RTA victims (*P* < 0.001) (Table 2). In terms of overall deaths, motorcycle RTA accounted for 54.0% of all deaths (128/237). However, 5.3% of victims had no information on their outcomes.

### 3.3. Factors Associated with RTA Mortality among Victims Admitted to Hospitals

Results from unadjusted and adjusted logistic regression models estimating mortality are reported in Table 3. Victims of other RTA, Glasgow Coma Score (GCS), year of occurrence (2017), victims' occupation (drivers), and study site (N'Zérékoré) were significantly associated with mortality in unadjusted models. However, after adjusting for covariates of interest, only GCS (mild or heavy/deep coma) and study site (N'Zérékoré) were independently associated with mortality. The likelihood of death increased with the GCS level. Compared to those with a normal GCS, victims in mild coma (AOR: 7.4; 95% CI: 2.1–25.8) and those in heavy or deep coma (AOR: 776.1; 95% CI: 340.2–1770.7) had increased odds of dying, respectively. In addition, victims of RTA in N'Zérékoré had increased odds of dying (AOR: 4.2; 95% CI: 1.6–11.2) compared with those in other cities excluding Siguiri.

## 4. Discussion

This study is the first to document the burden, morbidity, and mortality of motorcycle RTA in Guinea. We found that motorcycle RTA occur more frequently than other RTA in Guinea, affect a particular population, and are responsible for over half of RTA-related deaths. Death among all motorcycle RTA victims was significantly higher among those under 15 years, those aged 25 to 64 years, those in the N'Zérékoré district, and those with abnormal Glasgow Coma Scores at the entry to a hospital. These data suggest an urgent need for country-wide road safety planning and enforcement with additional attention to improving emergency care.

The observed predominance of motorcycle RTA in Guinea is consistent with other Africa studies reporting motorcycle RTA as the leading cause of RTA cases [17, 19, 21, 22]. The high burden of motorcycle RTA in Guinea may be related to the increasing importation of motorcycles from India and use of motorcycles in the country [7]. Furthermore, easy maneuverability of motorcycles, ability to travel on poor roads, and their ubiquitous use for inter- and intracity travel, as well as the conveyance of goods and services, probably play a role in the Guinean context [22–24]. In addition, the poor or nonexistent public transportation system has been reported to be an important contributing factor [21].

The most common injury types sustained by motorcycle RTA victims in our study were wounds and multiple injuries. Compared with studies from Cameroon, Tanzania, and Nigeria, the proportion of victims with wounds and multiple injuries in our study fell within the range (25.4%–65.9% and 21.7%–50.0%, respectively) [19, 21]. Though, a small proportion of motorcycle RTA victims died, they did account for over half of the overall deaths in this study.

We found that males, younger users, students, employees, and farmers/housewives suffered a significant burden of motorcycle RTA. Similar findings have been reported elsewhere in Africa [1, 8, 19, 22, 23], suggesting that the profile of motorcycle users is similar across the continent. The predominance of younger males and students in motorcycle RTA may be explained by the fact that they constitute the most mobile groups of the society, and they are the largest users of the road networks and they prefer motorcycles. Moreover, these categories are reported to be involved in high risk activities such as reckless and aggressive driving, impulsivity, and driving under the influence of alcohol [14, 25–28]. In addition, due to high unemployment in Guinea (5.2%, 2014) [15], it is possible that many young males and students engage in informal income-generating activities which include being a motorcyclist which increases their exposure to RTA risk. The fact that the most important economical age groups engage in motorcycle riding highlights an important economic consequence. For example, a cost estimate study reported that RTA cost USD $518 billion worldwide, comprising 1-2% of an individual country's annual Gross Domestic Product (GDP), which exceeds the total amount in development assistance that these countries receive [29, 30]. Another study estimating the population costs and effects of a selected set of enforcement strategies for reducing the burden of RTA injuries in developing countries reported that a combined enforcement of speed limits, drunk-driving laws, and motorcycle helmet use saves one disability-adjusted life years (DALY) for a cost of $1000–3000 [31]. Given this, conducting periodic campaigns at the community level and in schools to raise awareness about the risk of RTA and death in Guinea may be an important prevention strategy [32]. Additionally, regulations restricting the number of people allowed on a motorcycle including enforcement of these regulations may also help [33].

We observed a decrease in the numbers of motorcycle RTA every year from 2015 to 2017. This finding is consistent with that of the last yearbook of the country (2018) reporting a decrease of 9.4% in the number of RTA in 2017 compared to 2016 [15]. The decreasing trend in motorcycle RTA reported in this study might reflect the results of several community-based awareness raising campaigns that have been held around the country since 2015 [34, 35] or the influence of potential factors such as reduced access to care, under reporting, capacity issues, or competing disease burden at the hospital level. This finding therefore highlights the need for Guinea to consider improving the quality of collected data and using multiple sources of RTA data to help capture the real burden of the phenomenon [36].

Though motorcycle RTA were the commonest type of RTA in all the cities covered by this study, the cities of Boké and Siguiri accounted for the highest burden. Furthermore, the city of N'Zérékoré was independently associated with motorcycle RTA mortality. The high number of motorcycles in these cities may be due to the fact that they stand as the most vital mining and agricultural (N'Zérékoré) zones of the country, marked by a growing socioeconomic status of local population, allowing for increasing motorcycle ownership [15].

This study has some strengths. First, selecting six districts out of eight as study sites meant that the findings are representative of the burden of motorcycle RTA at the district level, apart from the fact that RTA victims were excluded because information on type of RTA was missing. Second, the study was conducted and reported according to the Strengthening the Reporting of Observational Studies in Epidemiology (STROBE) guideline [37]. However, the study findings should be interpreted in consideration of the limitations which include the retrospective design, the use of one source of RTA data, the high proportion of missing data, the fact we did not assess the causes of RTA involving our victims, our inability to disaggregate road users by the vehicle type (e.g., motorcycle vs. car) due to the structure of our secondary data source, and also scoring injury severity was not the primary purpose of this paper. Furthermore, some victims might have sought care elsewhere, and police records were not considered in this study. It is thus likely that the burden of motorcycle RTA was somewhat underestimated.

These data suggest that there is an urgent need for Guinea to focus on the application and enforcement of poor or insufficient road traffic legislation to help reduce the number of fatalities due to motorcycle RTA [1].

## 5. Conclusion

This study reported a high burden of motorcycle RTA and related mortality over three years in Guinea despite a slight linear decrease in the annual trend. Males, younger users, students, employees, and those residing in mining zones were the most affected. There is a need for stronger enforcement and application of road traffic safety laws, as well as targeted reporting labor-intensive injury severity scoring in the future study and awareness raising campaigns to prevent and control the phenomenon of motorcycle RTA in Guinea.

## Figures and Tables

**Table 1:** Sociodemographic characteristics of RTA victims admitted to hospitals by type of road traffic accidents, Guinea 2015–2017.

Variables	Motorcycle RTA (*N* = 11,623)		Other RTA (*N* = 3,339)		*P* value
	*N*	%	*N*	%	
*Sex*	*N* = 11,620		*N* = 3,336		<0.001
Male	8,536	73.5	2,121	63.6	
*Age*	*N* = 11,623		*N* = 3,339		<0.001
Children	2,036	17.5	452	13.5
Youth	4,168	35.9	929	27.8
Adults	4,973	42.8	1,817	54.4
Elderly	446	3.8	141	4.2
*Occupations*	*N* = 10,600		*N* = 3,083		<0.001
Students	3,150	29.7	612	19.8
Farmers/housewives	2,467	23.3	685	22.2
Employees	2,486	23.6	768	24.9
Sellers	1,284	12.1	605	19.6
Drivers	1,213	11.4	413	13.4
*Type of road users*	*N* = 4,949		*N* = 2,070		<0.001
Drivers	2,511	50.7	104	5.0
Passengers	1,954	39.5	1,848	89.3
Pedestrians	484	9.8	118	5.7
*Districts*	*N* = 11,623		*N* = 3,339		<0.001
Siguiri	3,559	85.6	597	14.4
Kindia	2,800	71.0	1,146	29.0
Mamou	1,753	71.1	714	28.9
Boke	1,335	86.5	208	13.5
Faranah	1,301	78.7	352	21.3
N'Zerekore	875	73.1	322	26.9
*Year*	*N* = 11,623		*N* = 3,339		0.002
2015	4,527	78.7	1,223	21.3	
2016	3,929	78.0	1,105	22.0	
2017	3,167	75.8	1,011	24.2	

RTA: road traffic accidents; *N*: total number for each variable.

**Table 2:** Clinical characteristics and outcomes of RTA victims admitted to hospitals by type of road traffic accidents, Guinea, 2015–2017.

Variables	Motorcycle RTA (*N* = 11,623)		Other RTA (*N* = 3,339)		*P* value
*Type of injury*	*N*	%	*N*	%	<0.001
No injury	1,670	14.4	800	24.0
Wounds	4,559	39.2	912	27.3
Fractures	165	1.4	85	2.6
Traumatic brain injury	138	1.2	80	2.4
Two or more injuries	5,091	43.8	1,462	43.8
*GCS*	(*N* = 11,528)		(*N* = 3,312)		<0.001
Normal	11,031	95.7	3,116	94.1
Mild coma	334	2.9	75	2.3
Heavy or deep coma	163	1.4	121	3.6
*Death*	(*N* = 11,623)		(*N* = 3,247)		<0.001
Yes	128	1.1	109	3.3
Not recorded	287	2.5	92	2.8

GCS: Glasgow Coma Score; *N*: total number for each variable.

**Table 3:** Logistic regression showing mortality predictors in victims of motorcycle and other types of road traffic accident who were admitted to a hospital in Guinea, 2015 to 2017.

Variables	Death			
	Unadjusted models OR (95% CI)	*P* value	Adjusted models AOR (95% CI)	*P* value
*Age*				
Children	0.8 (0.5–1.1)	0.161	2.2 (0.5–8.1)	0.285
Youth	0.5 (0.3–0.7)	<0.001	0.3 (0.1–0.8)	0.013
Adults	1	0.161	1	
Elderly	1.0 (0.5–1.7)	0.843	0.2 (0.0–1.5)	0.110
*Sex*				
Female	1		1	
Male	1.3 (1.0–1.9)	0.073	0.7 (0.3–1.7)	0.499
*Type of road users*				
Drivers	1		1	
Passengers	1.1 (0.7–1.7)	0.592	0.6 (0.2–1.6)	0.297
Pedestrians	1.7 (0.9–3.1)	0.087	0.8 (0.2–3.2)	0.701
*Occupations*				
Students	1		1	
Sellers	1.6 (1.0–2.5)	0.045	1.0 (0.3–3.7)	0.981
Farmers/housewives	1.3 (0.9–2.0)	0.188	0.4 (0.1–1.7)	0.232
Employees	1.4 (0.9–2.1)	0.150	1.0 (0.3–3.0)	0.991
Drivers	2.5 (1.6–3.8)	<0.001	0.8 (0.2–2.9)	0.791
*Type of injury*				
No injury	1		1	
Wounds	0.1 (0.0–0.2)	<0.001	0.2 (0.1–0.8)	0.027
Traumatic brain injury	2.0 (1.0–4.1)	0.057	0.6 (0.2–2.5)	0.489
Fractures	0.2 (0.0–1.4)	0.111	1	**—**
Two or more injuries	1.2 (0.9–1.6)	0.348	0.5 (0.2–1.2)	0.108
*GCS*				
Normal	1		1	
Mild coma	7.4 (3.7–14.7)	<0.001	7.4 (2.1–25.8)	0.002
Heavy or deep coma	425.1 (292.7–617.4)	<0.001	776.1 (340.2–1770.7)	0.000
*Type of RTA*				
Motorcycle RTA	1		1	
Other	3.0 (2.3–3.9)	<0.001	1.8 (0.7–4.3)	0.193
*Year*				
2015	1		1	
2016	0.7 (0.5–1.0)	0.030	0.6 (0.2–1.4)	0.215
2017	1.6 (1.2–2.2)	0.001	1.4 (0.7–2.8)	0.385
*Districts*				
Other	1		1	
N'Zérékoré	4.6 (3.4–6.3)	<0.001	4.2 (1.6–11.2)	0.003
Siguiri	0.9 (0.6–1.2)	0.414	0.7 (0.2–2.4)	0.514

OR: odds ratio; CI: confidence interval; AOR: adjusted odds ratio; GCS: Glasgow Coma Score.

## Data Availability

The data used to support the findings of this study are available from the corresponding author upon request.
